# Conceptualizing Human Microbiota: From Multicelled Organ to Ecological Community

**DOI:** 10.1155/2008/613979

**Published:** 2008-10-29

**Authors:** Betsy Foxman, Deborah Goldberg, Courtney Murdock, Chuanwu Xi, Janet R. Gilsdorf

**Affiliations:** ^1^Department of Epidemiology, University of Michigan, Ann Arbor, MI 48109, USA; ^2^Department of Ecology and Evolutionary Biology, University of Michigan, Ann Arbor, MI 48109, USA; ^3^School of Natural Resources and Environment, University of Michigan, Ann Arbor, MI 48109, USA; ^4^Department of Environment Health Sciences, University of Michigan, Ann Arbor, MI 48109, USA; ^5^Department of Pediatrics, University of Michigan, Ann Arbor, MI 48109, USA

## Abstract

The microbiota of a typical, healthy human contains 10 times as many cells as the human body and incorporates bacteria, viruses, archea, protozoans, and fungi. This diverse microbiome (the collective genomes of the microbial symbionts that inhabit a human host) is essential for human functioning. We discuss the unstated assumptions and implications of current conceptualizations of human microbiota: (1) a single unit that interacts with the host and the external environment; a multicelled organ; (2) an assemblage of multiple taxa, but considered as a single unit in its interactions with the host; (3) an assemblage of multiple taxa, which each interacts with the host and the environment independently; and (4) a dynamic ecological community consisting of multiple taxa each potentially interacting with each other, the host, and the environment. Each conceptualization leads to different predictions, methodologies, and research strategies.

## 1. INTRODUCTION

The scientific community has just begun to appreciate the number and complexity of organisms inhabiting the human body. The human microbiota contains 10 times as many cells as the human body and incorporates bacteria, viruses, archea, protozoans, and fungi. Many essential body processes require the presence of these diverse microorganisms to maintain pH in the oral and vaginal cavities, prevent invasion by pathogenic organisms, stimulate the immune system, aid digestion, and provide nutrients essential to our health. If a diverse microbiota is essential for human functioning [1], disruption of the normal microbiota should have significant negative consequences for human health. Indeed, studies suggest that the gut microbiota can influence risk of obesity [2], inflammatory bowel disease [3], cardiovascular disease [4, 5], and allergies and asthma [6].

The National Institutes of Health recently launched a series of initiatives focused on characterizing the human microbiome, the collective genomes of the microbial symbionts that inhabit a human host. Characterizing the microbiome provides insight into the diversity of genomes inhabiting the human host and is a first step towards understanding the complicated interactions among symbionts and between the symbionts and the human host. This launch has stimulated much discussion on why and how the human microbiome should be characterized. There has been little explicit discussion, however, of the underlying conceptualizations or models of the microbiota which might guide this characterization. Models provide a framework for designing experiments and for making inferences and predictions. In this commentary, we describe the range of conceptualizations of the human microbiota that have been implicit in different segments of this emerging literature. By making explicit the underlying models, we reveal the underlying assumptions and can consider the strengths and weaknesses of the different models in fitting existing observations, identify important data gaps, make predictions, and consider what model best applies in a given situation or for a given research or clinical question.

**Figure 1:**
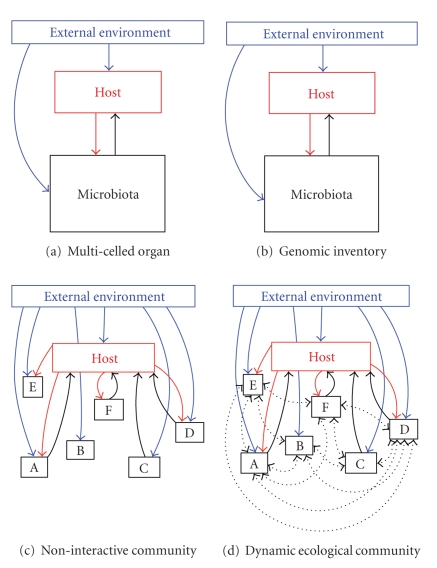
Four conceptualizations of human microbiota that focus to varying degrees on structure and/or function of the microbiota as a whole or of the component microbial taxa. Assumptions and implications of the extremes of simplicity and tractability on one hand (the multicelled organ conceptualization, Figure 1(a)) and complexity and relative intractability (the dynamic ecological community conceptualization, Figure 1(d)) are described in Table 1. All the interactions (linking arrows) are mediated to some extent by changes in the internal environment, which is not shown to enhance clarity. Mechanisms underlying the various interactions, including the role of internal environment, are depicted in Figure 2.

Figure 1 graphically displays the range of conceptualizations of the microbiota implicitly described in the following literature.

1. The microbiota considered as a single unit that interacts with the host and the external environment; a multicelled organ.
2. The microbiota consisting of multiple taxa, but considered as a single unit in its interactions with the host.
3. The microbiota as an assemblage of multiple taxa, which each can interact with the host and the environment independently.
4. The microbiota as a dynamic ecological community consisting of multiple taxa each potentially interacting with each other, the host, and the environment.

Conceptualizations (1) and (4) are clearly extremes and most research probably falls somewhere between them. However, because they are extremes, we can more clearly contrast them and their underlying assumptions, which have different implications for the development of clinical interventions (see Table 1). We expect an understanding of the human microbiota to require a melding of conceptualizations and associated theories before the promise of translating this understanding to new prevention, diagnostic, and treatment strategies can be achieved.

## 2. THE MICROBIOTA AS A MULTICELLED ORGAN

The microbiota is implicitly assumed to be much like a multicelled organ in much of the medical literature (Table 1) [2]: like an organ, a healthy microbiota consumes, stores, and redistributes energy and mediates important chemical transformations that benefit the host [7]. Communication among the cells that make up the microbiota enables replication and repair, and a set of feedback loops link host and microbiota (Figure 1(a)). The focus of an organ conceptualization is on *function*, with metabolic products and immune or neurological responses depending on the microbiota as a whole [7]. This view also implicitly assigns borders to the unit of interest, assuming that each spatially defined set of microbiota—the gut, oral community, or vaginal community—exists as a distinct and independent entity, and that each entity interacts with the host and the external environment as a single unit. Perhaps most importantly, this conceptualization assumes that any variation in the microbiota over time and between individual hosts is not functionally important or can be overlooked because of redundancies in genetic elements encoding various metabolic pathways in different strains or species. These unstated assumptions, summarized in Table 1, have the advantage of simplifying the system and focusing our attention on measuring inputs and outputs, physical structure, and defining spatial boundaries.

Conceptualizing the microbiota as an organ suggests research should characterize the range of inputs and outputs and immune response to the outputs and correlate them with healthy and diseased states for development of diagnostics. This conceptualization also implies that a therapeutic that adjusts the inputs and outputs could return the organ to a healthy state or substitute for a poorly functioning organ. For example, early diabetes—a malfunctioning pancreas—is diagnosed by measuring organ inputs (glucose levels), and is treated by decreasing inputs (lowering glucose levels) or supplying output (insulin). We might envision similar inputs and outputs that can be used to diagnose and correct disrupted microbiota in the skin, mouth, gut, or vaginal cavity.

Assuming a physical structure and boundaries stimulates studies to explore that structure and define boundaries. For example, conceptualizing the microbiota as an organ leads us to consider that the microbiota on the skin or intestinal lumen might form physical structures, such as biofilms. This structure might vary in size and composition, being a thick lawn in some areas and thin islands in others and act as an additional physical barrier to colonization by pathogens. Disrupting these protective biofilms chemically or physically may lead to invasion by pathogens. Additionally, the size or denseness of the structure might in some surfaces be associated with disease. Assuming a defined boundary suggests that microbiota might be moved or be removed, and that there are optimal areas for measuring inputs and outputs. These are all testable hypotheses. The disadvantage of conceptualizing the microbiota as an organ is that it necessarily minimizes the complexity of a diverse microbiota, which may lead us to either underestimate the possible unintended consequences or overestimate the potential of proposed interventions.

The current research focus on cataloging the diversity of microbiota using genomic techniques [8] takes a step beyond viewing the microbiota as a single, homogeneous unit (Figure 1(b)). While a critical next step, this approach goes no further than the basic organ-view in understanding the *mechanisms* that drive variation in function of the microbiota; the underlying assumptions and implications of this approach remain quite similar to those of the “microbiota as organ” conceptualization (Table 1).

## 3. MICROBIOTA AS AN ECOLOGICAL COMMUNITY

The other extreme is to conceptualize the microbiota as a continuum of dynamic ecological communities living in the numerous microhabitats of the human body [9]. Each species or strain of the microbiota interacts with other members of the microbiota and with the host, as well as with the external environment (Figure 1(d)). This conceptualization highlights interactions between component organisms and their dynamics; a dynamic and spatially continuous system is assumed, and the net effects can be positive, negative, or neutral towards the host (Table 1). Key to this conceptualization is that understanding the underlying processes that control community structure, including the interactions among the microbiota themselves, is essential for understanding its function. This conceptualization has the advantage of increased realism, but is much more complex and consequently may be less useful for some purposes.

Considering microbiota as an ecological community stimulates research into how that community reacts to insults. For example, a number of conditions, such as reactive arthritis, occur in some individuals in response to infection. One current theory is that certain microbial surface antigens mimic host cell receptors, so individuals with a particular variant in immune signals generate an immune response to their own cells after infection has cleared. The role of microbiota in mediating this response has not yet been considered. However, we know that the gut microbiota is important in modulating host immune response [2]. It is possible that bacteria that lead to reactive arthritis disrupt the signals between the human body and the microbiota such that the immune system no longer sees organisms with antigens similar to those of the host as self, leading to self-attack. Consequently, the reason that reactive arthritis is frequently self-limiting may be related to restoration of the normal microbiota with subsequent restoration of immune signals.

While some research has conceptualized the human microbiota as an ecological community, the interactions among microbiota remain almost completely unexplored [10]. Most work is similar to the conceptualization in Figure 1(c), characterized by independent relationships between each member of the microbiota and its human host, but not among the microbiota themselves [5]. However, we suspect that interactions among members of a community, including the numerous indirect pathways of influence generated in such webs, are integral to understanding the dynamic and spatially heterogeneous nature of many aspects of the human microbiota and, therefore, to the functioning of those communities [11–13]. If so, however, complex and difficult, research must address how this understanding of ecological dynamics and function can be translated to successful clinical interventions.

## 4. RESEARCH AND CLINICAL CONSEQUENCES OF ALTERNATIVE CONCEPTUALIZATIONS OF THE HUMAN MICROBIOTA

**Figure 2:**
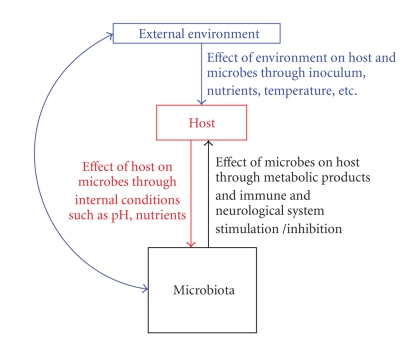
Potential mechanisms of interactions between external environment, host and the microbiota in the multicelled organ conceptualization of human microbiota.

The underlying conceptualization of the microbiota guides, either explicitly or implicitly, medical approaches to treating and preventing conditions of disrupted flora. An organ view assumes that switching from an unhealthy (dysfunctional) to a healthy (functional) state can be achieved by manipulating inputs or outputs. With this model in mind, the associated research agenda will focus on characterizing the products of the microbiota, their healthy and diseased ranges, and how the products are affected by host characteristics and the external environment (Figure 2). Therapeutic studies will seek to shift metabolic products or cell signals back to the functional state associated with health.

By contrast, if we conceptualize the microbiota as multiple communities of interacting genomes, we might instead try to reestablish or maintain a specific microbial community structure associated with health. Success of this approach depends on reestablishing a healthy microbial community, with all its associated feedbacks. The fact that we currently lack sufficient understanding to establish complex ecological communities with a full complement of functioning interactions may account for disappointing and inconsistent results when probiotics have been used to treat vulvovaginal candidiasis and antibiotic-associated diarrheas: merely adding organisms to a complex system—even in large amounts—can be insufficient to lead to a healthy community structure [14, 15].

## 5. INTEGRATING THE CONCEPTUALIZATIONS: FUTURE RESEARCH ON THE HUMAN MICROBIOTA AND HEALTH

The Human Microbiome Project (HMP) is a major roadmap initiative of the National Institutes of Health (NIH) [8]. Each NIH institute has been exploring various ways to meet the goals of the initiative, primarily from an organ viewpoint, in keeping with the organization of the institutes by disease or organ system. As the HMP moves forward, it would benefit from the development of an overall conceptual framework for structuring the research agenda, analyzing the resulting data, and applying the results in order to improve human health. Given the complexity of interactions among organisms in the human microbiota and the complexities and variations of human hosts and the organisms that inhabit those hosts, a catalog of microbes even from a range of multiple, diverse, individuals is only a first step towards the ultimate goal of manipulating human microbiota to prevent and treat disease. Further progress will require understanding the *drivers* of change in human microbiota that lead to disease states, particularly the underlying mechanisms and functions of microbiota, and how to establish and maintain communities consistent with health. Understanding the mechanisms and functions that process inputs and lead to outputs will enhance our ability to consistently manipulate the microbiota in the form of medical interventions and to minimize the unintended consequences of those interventions.

The level of complexity required to take a dynamic ecological view of human microbiota is daunting and will require collaborations among many disciplines including molecular biology, ecology, medicine, epidemiology, and mathematics. To fully understand the mechanisms that drive community structure and function, microbiota must be examined over time to determine the dynamics of its processes and over space to determine the interconnectedness of microbiota within an individual host and the range of microbiota among individuals. A comparison of microbiota among individuals living in countries with poorer sanitation to those with high levels of sanitation might be particularly interesting, in that normal, healthy, microbiota from less developed areas may regularly include helminthes. Moreover, these studies will require testing large numbers of diverse individuals, as the range of what is “healthy” or “normal” is probably very wide and may depend, in part, on the genetic make-up of the host and the associated environment. In addition, experimental approaches will be essential to interpret descriptive studies. Experiments in well-controlled model systems such as bioreactors or animal models will be useful to isolate subsets of the interacting components depicted in the dynamic ecological community model (Figure 1(d)). Such experiments will provide a critical bridge between descriptions of highly diverse communities that change over time and space on one hand and the logistically intractable task of experimental investigation of all possible interaction pathways in such communities. Isolating key components of communities for intensive study of interactions has been very successful in understanding the ecology of macrocommunities [16–19]. Finally, mathematical models that require specification of the hypothesized underlying systems will enable conduct of simulation experiments to understand direct and indirect effects. The validity of simulation experiments depends heavily on the data available to “dock” the model. All of these approaches should lean heavily on well-developed ecological and evolutionary theories to form hypotheses and testable, quantifiable predictions.

Neither of the two extreme conceptualizations of the human microbiota, the multicelled organ and the ecological community model, are likely to be the most useful; integrated conceptualizations may be most appropriate for different research questions or clinical problems. Regardless of our conceptualization, however, we need to recognize that implicit assumptions yield different predictions on the impact of microbiota function on human health and move the research agenda in different ways. As the biomedical community moves into this rapidly burgeoning area, funds should be set aside to explore and develop theoretical underpinnings that draw on existing ecological and evolutionary theories and, thus, hasten efforts towards the ultimate goal of maintaining a healthy microbiota to maintain human health.

## Figures and Tables

**Table 1:** Underlying assumptions of conceptualizing human microbiota as a multicelled organ versus an ecological community. Some of the assumptions of the multicelled organ conceptualization also apply to the intermediate conceptualizations depicted in Figure 1.

	Multicelled organ	Ecological community
Assumptions	(1) Identification of component microbes is not necessary for prediction of function	(1) Understanding interactions among microbiota is essential to predict function
	(2) Metabolic products and immune responses are characteristic of the microbiota as a whole	(2) Metabolic products and immune responses are a consequence of community structure and microbial interactions
	(3) Static (changes in healthy microbiota over time are not functionally important)	(3) Dynamic
	(4) Boundaries exist (movement of microbes is not important)	(4) Spatially continuous and linked by immigration and emigration
	(5) Host-to-host variation in microbiota is not important	(5) Host-to-host variation is functionally important
	(6) Microbiota functions for benefit of the host	(6) Net microbiota effects can range from negative to neutral to positive

Implications	(1) Healthy microbiota function is evaluated by its metabolic products and immune responses	(1) Healthy microbiota function is evaluated by both microbial community structure and its metabolic products and immune responses
	(2) Health is restored by providing the right signals/products that are missing or by neutralizing negative signals/products	(2) Health is restored by shifting the community and component interactions, which requires an understanding of processes that control community structure and interaction webs
	(3) Appropriate therapies include broad-spectrum antibiotics, microbiota transplants, direct manipulation of metabolic products, or immune signals	(3) Appropriate therapies include carefully tailored probiotics, modification of internal, or external environment to modify specific interactions
